# Trade-off between Gradual Set and On/Off Ratio in HfO_*x*_-Based Analog Memory with a Thin SiO_*x*_ Barrier Layer

**DOI:** 10.1021/acsaelm.3c00131

**Published:** 2023-06-01

**Authors:** Fabia F. Athena, Matthew P. West, Jinho Hah, Samuel Graham, Eric M. Vogel

**Affiliations:** † School of Electrical and Computer Engineering, Georgia Institute of Technology, Atlanta, Georgia 30332, United States; ‡ School of Materials Science and Engineering, Georgia Institute of Technology, Atlanta, Georgia 30332, United States; § Department of Mechanical Engineering, University of Maryland, College Park, Maryland 20742, United States; ∥ George W. Woodruff School of Mechanical Engineering, Georgia Institute of Technology, Atlanta, Georgia 30332, United States

**Keywords:** neuromorphic computing, graduality, on/off ratio, barrier-layer, interface, FEA simulation

## Abstract

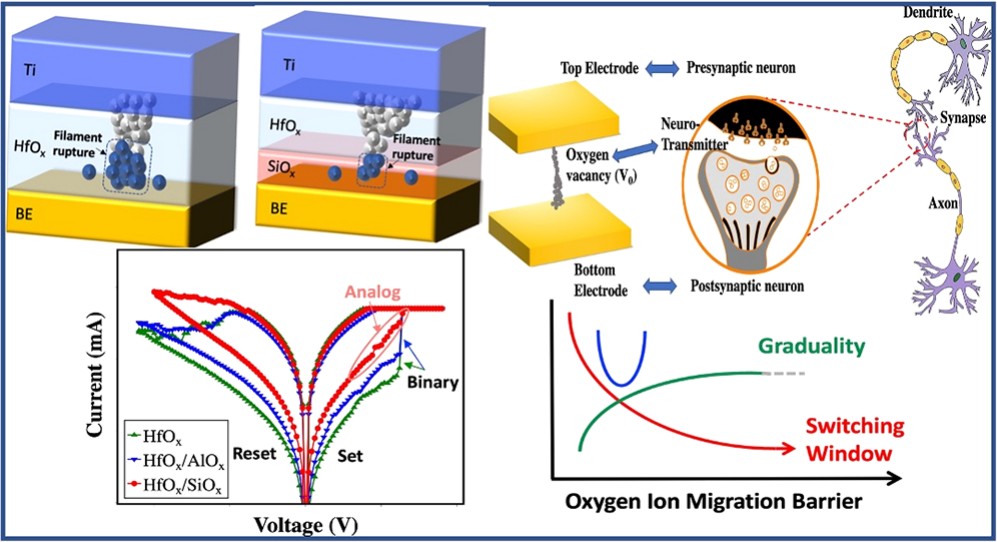

HfO_*x*_-based synapses are widely accepted as a viable candidate for both in-memory and neuromorphic computing. Resistance change in oxide-based synapses is caused by the motion of oxygen vacancies. HfO_*x*_-based synapses typically demonstrate an abrupt nonlinear resistance change under positive bias application (set), limiting their viability as analog memory. In this work, a thin barrier layer of AlO_*x*_ or SiO_*x*_ is added to the bottom electrode/oxide interface to slow the migration of oxygen vacancies. Electrical results show that the resistance change in HfO_*x*_/SiO_*x*_ devices is more controlled than the HfO_*x*_ devices during the set. While the on/off ratio for the HfO_*x*_/SiO_*x*_ devices is still large (∼10), it is shown to be smaller than that of HfO_*x*_/AlO_*x*_ and HfO_*x*_ devices. Finite element modeling suggests that the slower oxygen vacancy migration in HfO_*x*_/SiO_*x*_ devices during reset results in a narrower rupture region in the conductive filament. The narrower rupture region causes a lower high resistance state and, thus, a smaller on/off ratio for the HfO_*x*_/SiO_*x*_ devices. Overall, the results show that slowing the motion of oxygen vacancies in the barrier layer devices improves the resistance change during the set but lowers the on/off ratio.

## 1 Introduction

Traditional artificial neural networks based on von-Neuman computing consume significant energy and are facing challenges for data-intensive tasks due to the physical separation of memory and logic processing.^1,2^ Taking inspiration from biological cognition, neuromorphic computing aims to enable artificial intelligence to perform closer to biological cognition for complex tasks such as self-driving, language translation, pattern and speech recognition, and real-time health monitoring.^3−10^

Two-terminal memristors,^11,12^ which use adaptive oxides such as TiO_*x*_,^13^ HfO_*x*_,^14^ TaO_*x*_,^15^ AlO_*x*_,^16^ and NiO^17^ as the active layer, exhibit a change in resistance with the application of bias and are being considered for both non-volatile in-memory and brain-inspired neuromorphic computing. Among these, HfO_*x*_ devices are attractive because of their CMOS compatibility, scalability (<10 nm), fast switching (∼ns), excellent switching endurance (>10^10^ cycles), and data retention (10 years).^18^ Although HfO_*x*_-based resistive random-access memory appears promising for non-volatile memory, it is not fully optimized for neuromorphic computing.^18^ To achieve ideal analog neuromorphic computing, a synapse device should possess multiple qualities, such as >10 years non-volatility, <10 nm scalability, >5 bit analog memory, large on/off ratio, and so forth.^19^ In addition to these, gradual and symmetric resistance changes and less device–device variability are the major requirements to improve the pattern recognition accuracy of deep learning models.^19,20^ Highly abrupt and stochastic resistance changes of existing memristor technologies make it challenging to apply stochastic gradient descent for error calculation in deep learning.^21^

Previous studies have achieved controlled resistance change in memristor devices using non-CMOS compatible materials, such as Ag, ZnO, and Ge, or circuitry-based solutions. For instance, a Ge-implanted SiN_*x*_/a-Si memristor showed gradual resistance change by inducing structural defects in the a-Si layer.^22^ An Ag-Cu^23^ and co-sputtered Ag and Si memristor^24^ also showed controlled resistance change. Chandrasekaran et al.^25^ demonstrated that gradual resistance change and improved training epoch are achieved in the ZnO memristor. While the deposition methods used in these studies, such as evaporation and sputtering, are appropriate for demonstration purposes, atomic layer deposition (ALD) is more compatible with large-scale circuit fabrication.^26^ Therefore, it is necessary to achieve controlled resistance change in ALD-deposited oxides that are CMOS compatible.

Filamentary memristors based on CMOS-compatible oxides such as HfO_*x*_, AlO_*x*_, and TaO_*x*_ have been reported in past studies. Achieving controlled resistance changes during set with less variability in resistance change in filamentary memories is challenging. In the HfO_*x*_ filamentary device, a *V*_o_-rich conductive filament (CF) is created in the active layer (HfO_*x*_) during forming, and in the subsequent steps, the motion of a few oxygen vacancies (*V*_o_) out and into the CF causes reset and set, respectively. Typically, the reset is gradual because the resistance increases; as the bias becomes more negative, the temperature decreases, causing the resistance to change more slowly. The set is abrupt, which means that there is a sudden increase of current with bias application. This happens because with an increasing positive bias, the resistance decreases, causing the temperature to increase and a sudden motion of *V*_o_ into the CF due to a positive feedback loop.^27−29^ Therefore, achieving controlled resistance change during the set in HfO_*x*_ filamentary memristors has been a critical challenge. Wu et al.^30^ reported that the addition of a TaO_*x*_ electrothermal modulation layer in a HfO_*x*_-stacked device helps achieve controlled resistance change during set by enhancing the thermal environment and forming multiple weak CFs. However, the applied voltage was high, and because of the multiple weak CFs, the variability in resistance change was large. Some studies^31,32^ reported that adding an AlO_*x*_ layer at the bottom-electrode/oxide interface in the HfO_*x*_ device slows down the motion of *V*_o_ because of the high migration barrier of *V*_o_ in the AlO_*x*_ layer. The slow motion of *V*_o_ leads to controlled resistance change during set. However, these studies do not include any analysis of variability among the results of multiple devices. As each of these examples show, achieving controlled resistance changes with less variability in resistance change is still necessary. Moreover, in AlO_*x*_ barrier layer devices, the gradual set was achieved at the expense of the on–off ratio. The fundamental reason behind the trade-off between the on–off ratio and the gradual set is unknown. Additionally, the gradual set benefit depends on the thickness of the barrier layer, as a recent study^33^ reported that HfO_*x*_ with 0.5 nm AlO_*x*_ barrier layer shows an abrupt set. Therefore, the investigation of CMOS-compatible barrier layer material of optimum thickness for controlled resistance change during set and the concurrent impact on the on/off ratio is necessary.

In this paper, the effect of a thin (∼1 nm) barrier layer (e.g., SiO_*x*_, AlO_*x*_) on the switching of ALD-deposited HfO_*x*_ memristors is evaluated. The elemental distribution is characterized using X-ray photoelectron spectroscopy (XPS) depth profiling. The barrier layer is added to the oxide/BE electrode interface, where the CF breaks during reset.^34−39^ Compared to AlO_*x*_ (1.26–3.6 eV)^40,41^ and HfO_*x*_ (0.7–1.5 eV),^27,42,43^ the SiO_*x*_ barrier layer exhibits a high activation energy for oxygen vacancy diffusion, ranging from 2.03–4.6 eV.^44−48^ This makes it a more formidable oxygen vacancy migration barrier than both AlO_*x*_ and HfO_*x*_. This is expected to enable controlled resistance change during the set due to the slower motion of *V*_o_. The HfO_*x*_/SiO_*x*_ devices exhibit controlled resistance changes during the set and less device–device, cycle–cycle variability. The on–off ratio (∼10) for memristors with HfO_*x*_/SiO_*x*_ oxides is still sufficient for many neuromorphic circuits but is lower than those with HfO_*x*._ To explore the relationship between the barrier layer addition and the low on–off ratio, a Finite Element Analysis simulation is performed. It confirms that a narrower oxide region is formed in the CF during the reset of the HfO_*x*_/SiO_*x*_ device, leading to a lower off-resistance state.

## 2 Experimental Details

### 2.1 Device Fabrication

**Figure 1:**
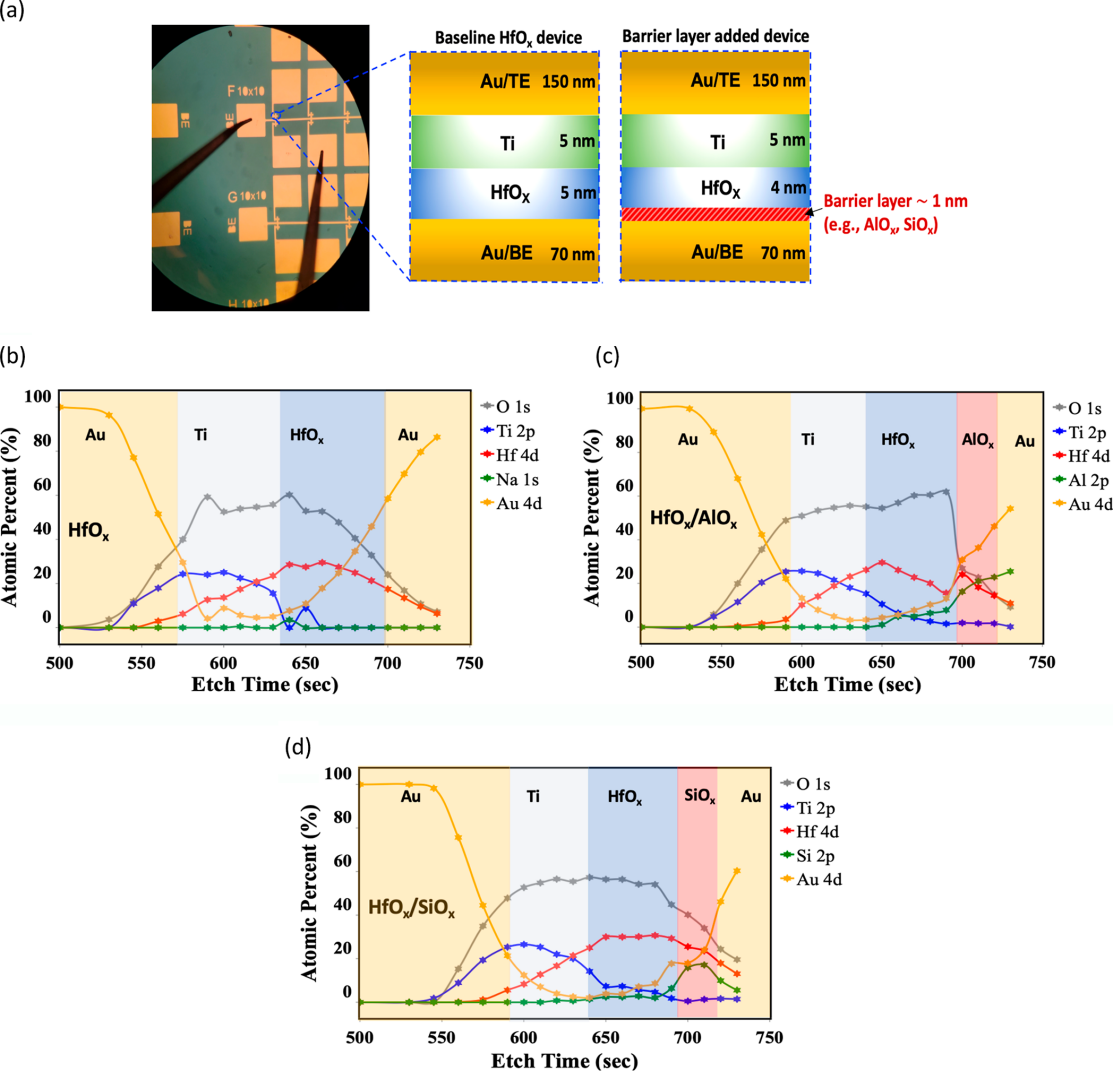
(a) Micrograph of a representative device and schematics of the unit cell of an oxide memristor with and without the barrier layer oxide. The AlO_*x*_ and SiO_*x*_ barrier layer oxides are used. XPS depth profiling shows the interfaces of the fabricated device structures. (b) HfO_*x*_ device, (c) HfO_*x*_/AlO_*x*_ device, and (d) HfO_*x*_/SiO_*x*_ device. The depth profile shows that the resulting structures have the barrier layer at the bottom Au electrode/oxide interface.

Metal–insulator–metal memristors with HfO_*x*_, HfO_*x*_/AlO_*x*_, and HfO_*x*_/SiO_*x*_ amorphous oxides were fabricated, as shown in Figure 1a. The SiO_2_/Si wafers with 308.5 nm of SiO_2_ were cleaned with acetone, methanol, and isopropanol. Bottom electrodes (BE) with a size of 10 × 10 μm^2^ were formed using mask-less ultraviolet photolithography followed by lift-off using acetone. Negative photoresist NR-9 was used to pattern the BEs. The BEs consisted of a ∼20 nm titanium adhesive layer and ∼70 nm of gold deposited using electron beam evaporation at a rate of 0.1 nm/s at a pressure of 2.62 × 10^–6^ Torr without breaking the vacuum using the Denton Explorer E-beam Evaporator. The active layer oxides were deposited in a Cambridge Nanotech Plasma ALD system at 250 °C. For the standard HfO_*x*_ device, the ∼5 nm HfO_*x*_ active layer was synthesized via thermal ALD of ∼55 cycles of tetrakis(dimethylamido) hafnium (TDMAHF) and deionized water precursors. For the HfO_*x*_/AlO_*x*_ devices, ∼1 nm AlO_*x*_ was synthesized using thermal ALD consisting of 9 cycles of trimethylaluminum (TMA) and deionized water followed by 44 cycles of TDMAHF and deionized water for ∼4 nm HfO_*x*_. Similarly, for the HfO_*x*_/SiO_*x*_, devices ∼1 nm SiO_*x*_ were synthesized via plasma-enhanced atomic layer deposition (PEALD) using 11 cycles of tris(dimethylamino)silane (3DMAS) and oxygen (50 sccm) followed by thermal ALD of 44 cycles of TDMAHF and deionized water for ∼4 nm HfO_*x*_. Specifically, the ∼1 nm SiO_*x*_ layer was deposited using the PEALD due to its compatibility with the 3DMAS precursor and ability to produce SiO_*x*_ films with high density and low impurity content at the processing temperature of 250 °C.^49−51^ Spectroscopic ellipsometry was utilized to verify the thicknesses of the oxide stacks. The ∼5 nm titanium capping layer and ∼150 nm gold top electrodes (TEs) were deposited using electron beam evaporation. For TE lithography, MF19-positive photoresist was used. For etching Au TEs, the devices were immersed in Transene gold etchant (TFA) for ∼45 s. Next, the remaining 5 nm Ti was etched using the standard etching method (at a gas flow rate of CHF_3_ 45 sccm, O_2_ 5 sccm, Ar 0 sccm, RF power of 250 Watts RF, and pressure of 40 mT, time = 60 min) via Vision Reactive Ion Etching (RIE). The devices were placed in an acetone bath for 4 h to remove the residual photoresist. Finally, cleaning was performed using sequential emersion in acetone, methanol, and isopropanol. In addition, before the BE and active layer oxide deposition, an oxygen plasma descum process was performed for 30 s (at a flow-rate of 50 sccm, plasma power of 150 Watts RF, and pressure of 60 mT) to remove the residual photoresist and surface hydrocarbons.

### 2.2 Electrical Characterization

A Keithley 4200 SCS semiconductor parameter analyzer was used to conduct the digital and analog electrical testing. The devices were formed by applying a positive voltage sweep at the top electrode with a current compliance of 0.1 mA. After forming, incremental negative reset voltages were applied from 0 V to the maximum achievable negative voltage with an increment of −0.1 V. This gradual reset process is performed to ensure that the filament rupture process occurs in a controlled way. The reset step was followed by the application of 30 hysteresis loops with a predefined set and reset maximum stop voltage. For each electric testing condition, the measurement was repeated on ∼8 devices to determine aggregate device statistics like standard deviation and mean.

### 2.3 Material Characterization

XPS was performed using a monochromatic Al K-alpha X-ray source (h υ = 1486.6 eV), 400 μm spot size, and 15 W X-ray gun power. The elemental distribution from the top gold electrode to the bottom gold electrode was characterized using XPS depth profiling. To avoid preferential sputtering in the XPS depth profiles, Ar^+^ sputtering was performed at an incident angle of 30° and low energy of 1 KeV ion energy.^52^ To correct the surface potential variation associated with charging, the C 1s peak at 285.0 eV was used as the reference energy. The XPS spectra were fitted using a Shirley background^53^ and Lorentzian–Gaussian (GL 30) line shapes with CASA XPS software.^54^

## 3 Results and Discussion

Figure 1a shows a microscopic view and schematic of the fabricated HfO_*x*_ synaptic device with and without the barrier layer. An optical viewgraph of a representative device is shown in Figure S1a,b. The device consists of a titanium capping layer and an active layer oxide (here, HfO_*x*_, HfO_*x*_/AlO_*x*_, and HfO_*x*_/SiO_*x*_) sandwiched between the top and bottom gold electrodes. The titanium capping layer is known to improve device characteristics such as nonvolatility at high temperature (85 °C), uniformity in switching and low forming voltage.^55,56^ During the forming operation, oxygen vacancy-rich conductive filaments are formed within the active layer oxides. While there is a range in general for the activation energy of oxygen vacancy diffusion,^41,47^ it is higher in SiO_*x*_ (2.03–4.6 eV)^44−48^ compared to AlO_*x*_ (1.26–3.6 eV)^40,41^ and HfO_*x*_ (0.7–1.5 eV).^27,42,43^ A depth profile of the elemental composition obtained using XPS is shown in Figure 1b–d for HfO_*x*_, HfO_*x*_/AlO_*x*_, and HfO_*x*_/SiO_*x*_, respectively. The XPS depth profiles show that the resulting interfaces of the device structures have SiO_*x*_ and AlO_*x*_ barrier layers at the bottom electrode interface. The thicknesses of the barrier layers are ∼1 nm, which is confirmed by ellipsometry analysis in Figure S1c.

**Figure 2:**
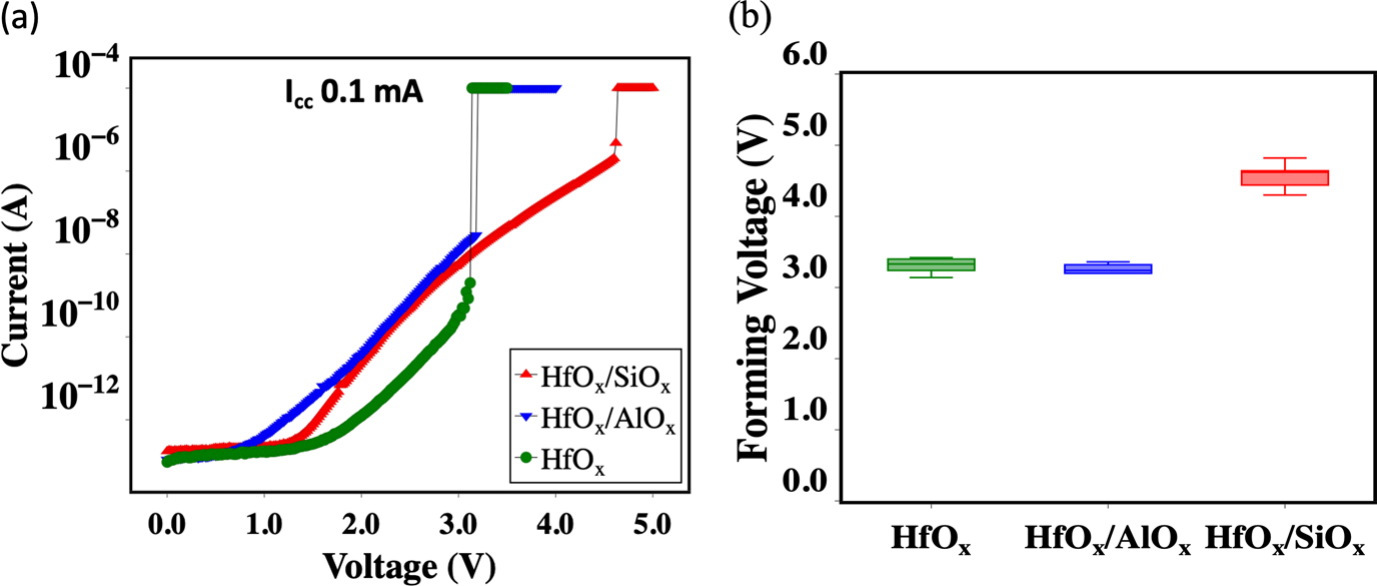
(a) Forming characteristics of the fabricated devices. Forming characteristics of HfO_*x*_/SiO_*x*_ are very different from the HfO_*x*_ and HfO_*x*_/AlO_*x*_ devices. (b) Statistics of the forming voltages. Forming voltages in HfO_*x*_ and HfO_*x*_/AlO_*x*_ are the same; however, forming voltages in HfO_*x*_/SiO_*x*_ devices are high (∼4.5 V).

Figure 2a shows typical current–voltage distribution of forming of the HfO_*x*_, HfO_*x*_/AlO_*x*_, and HfO_*x*_/SiO_*x*_ devices. Figure 2b shows that the average forming voltage of the HfO_*x*_ device (∼3.1 V) is similar to previously reported results for 5 nm HfO_*x*_ RRAM.^9,57^ The HfO_*x*_/AlO_*x*_ devices have the same average forming voltage as HfO_*x*_ devices. However, the forming voltage of the HfO_*x*_/SiO_*x*_ devices is higher (∼4.5 V). The forming process involves the creation of vacancies and the hopping of electrons through them, which increases the temperature and moves the vacancies. This process finally forms vacancy-rich filament(*s*) in the active layer oxide.^36^ Therefore, oxygen vacancy (*V*_o_) formation energy and migration barrier in the oxide directly govern the forming process. A high *V*_o_ migration barrier^44^ and formation energy in the SiO_*x*_ layer compared to HfO_*x*_ suggests a conical filament with a thicker section in HfO_*x*_ and a thinner section in the SiO_*x*_ layer in the HfO_*x*_/SiO_*x*_ devices. Wu et al.^39^ reported experimental evidence of conical filament due to migration barrier differences in HfO_*x*_ and SiO_*x*_ in the Ni/HfO_*x*_/SiO_*x*_ conductive bridge RRAM (CBRAM) stack, where the Ni-rich filament can be easily observed. The study showed an in situ high-resolution transmission electron microscopy (HRTEM) image of a conical filament having a narrow part in the SiO_*x*_ and the wider part in the HfO_*x*_. Previous studies have also reported that the shapes and stability of conductive filaments are directly correlated to the forming process in HfO_*x*_-based devices.^58^ Therefore, it is hypothesized that in HfO_*x*_ and HfO_*x*_/SiO_*x*_ devices, a significant difference in forming causes a difference in filament characteristics, which further results in different switching characteristics.

**Figure 3:**
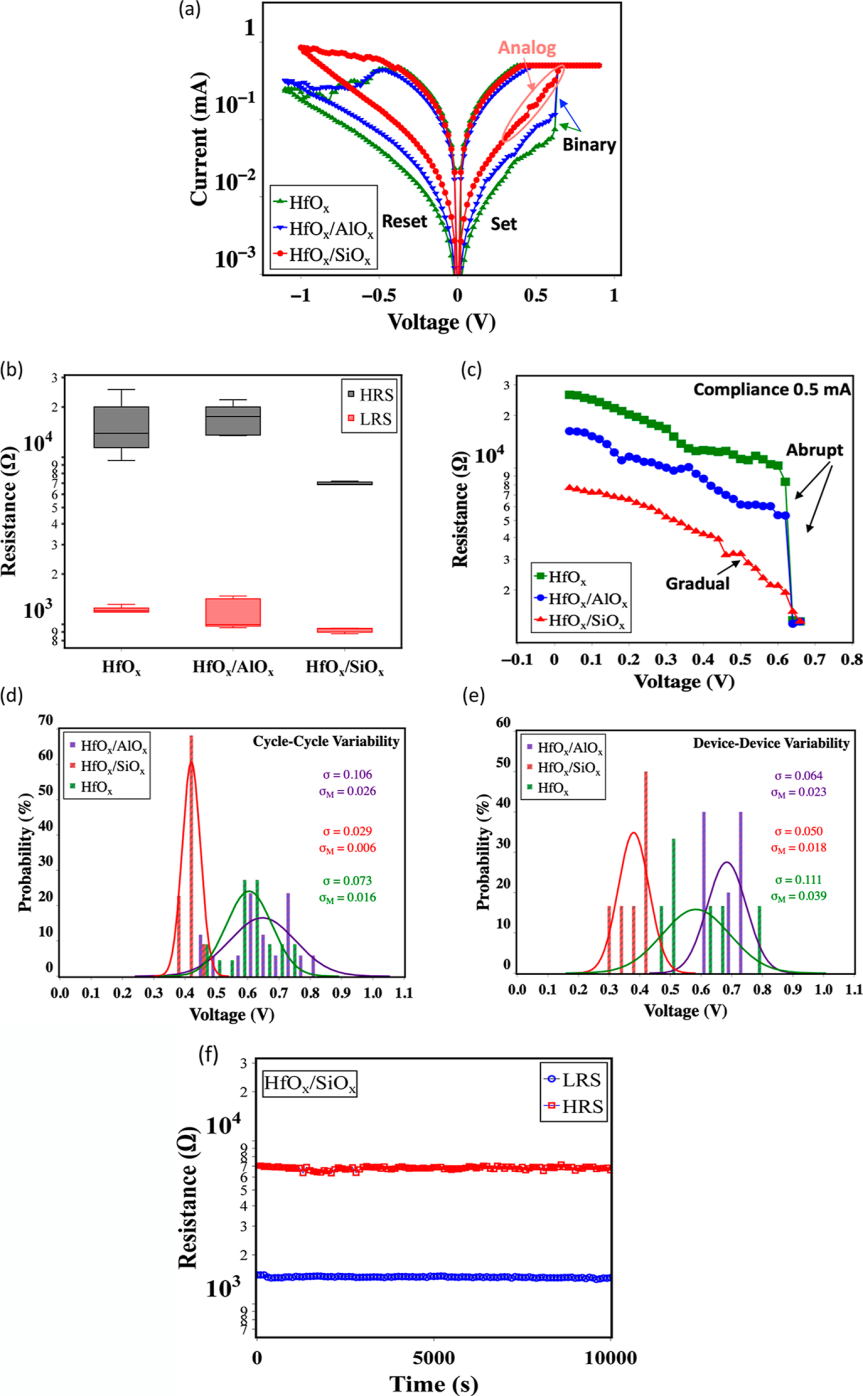
(a) Typical current–voltage relationship during the set and reset of the devices upon filament stabilization with >20 stabilization cycles. The set current compliance (*I*_cc_) is 0.5 mA. The HfO_*x*_/SiO_*x*_ synaptic device shows controlled resistance change during the set. The HfO_*x*_ and HfO_*x*_/AlO_*x*_ devices show abrupt set behavior (b) high resistance states (HRS) and low resistance states (LRS) in the devices. The switching window in HfO_*x*_/SiO_*x*_ devices is small compared to the HfO_*x*_ devices. The switching window decreases with the increase of the oxygen vacancy migration barrier. (c) Corresponding resistance changes from HRS to LRS during the set show an abrupt resistance change for both HfO_*x*_ and HfO_*x*_/AlO_*x*_ devices at 0.5 mA *I*_cc_. At the same compliance level, the HfO_*x*_/SiO_*x*_ device does not exhibit the abrupt resistance change observed in the other devices. Normal probability distribution plot of the set transition voltage showing (d) cycle-to-cycle variation and (e) device-to-device variation of the oxides. The set transition voltage is defined as the voltage at which the current reaches 0.1 mA. Here, σ is the standard deviation, and  is the standard error. The HfO_*x*_/SiO_*x*_ device exhibits an improvement in the standard deviation and the standard error compared to HfO_*x*_ and HfO_*x*_/AlO_*x*_. (f) Retention characteristics of the HfO_*x*_/SiO_*x*_ barrier layer device.

Figure 3a,b shows the digital switching characteristics and the high resistance state (HRS) and low resistance state (LRS) distribution of the devices. The baseline HfO_*x*_ devices show a sudden increase of current during positive bias application (abrupt set) and a slower current decrease during negative bias application. The HfO_*x*_/AlO_*x*_ device also has an abrupt set similar to HfO_*x*_. However, it has a decreased switching window, which is the ratio of the HRS to the LRS. In the HfO_*x*_/SiO_*x*_ device, the current change during the set is not abrupt, with the same compliance (∼0.5 mA). The relatively gradual change in current during set in HfO_*x*_/SiO_*x*_ device translates to a controlled resistance change as a function of positive bias, as shown in Figure 3c. On the contrary, an abrupt resistance change, demonstrating non-linearity, is observed in baseline HfO_*x*_ devices. The linearity in the *I*–*V* curve of non-volatile memory devices is crucial for precisely reading the current, which determines the results of an arithmetic operation in cross-point RRAM array.^59^ Prior studies have directly correlated the importance of *I*–*V* linearity with improved accuracy in image classification during neural network training.^60^ In addition, compared to HfO_*x*_ devices, the HfO_*x*_/SiO_*x*_ devices show less abrupt resistance change at a high compliance level (∼1 mA *I*_cc_), as shown in Figure S2a. However, the improvement is less compared to the ∼0.5 mA compliance level. At higher compliance levels, the reduction of *I*–*V* linearity is also observed in prior studies.^61^ In addition, to explore the variation in the device data, a statistical measurement of device–device and cycle–cycle variability is conducted on randomly chosen ∼ eight devices from each oxide sample. It is observed that HfO_*x*_/SiO_*x*_ devices have a lower cycle-to-cycle and device-to-device variation of the set transition voltage (*V*_set_)^23^ than the HfO_*x*_ devices, as shown in Figure 3d,e. The set transition voltage is voltage at which the current reaches ∼0.1 mA. The cycle–cycle standard deviation for HfO_*x*_/SiO_*x*_ device is observed to be ∼60% lower than the HfO_*x*_ device and ∼73% lower than the HfO_*x*_/AlO_*x*_ device, while the cycle–cycle standard error for the HfO_*x*_/SiO_*x*_ device is also observed to be more than 60% lower than both devices. The device–device variation also shows significant improvement, having ∼55 and ∼21% lower standard deviation than HfO_*x*_ and HfO_*x*_/AlO_*x*_ devices, respectively, with an identical improvement in the standard error. It is difficult to achieve both controlled resistance change and less variability in resistance change in filamentary RRAM.^30^ The relatively gradual resistance change in the HfO_*x*_/SiO_*x*_ device is also retained for a long time, as reflected in the current–voltage relationship measured after ∼6000 h of the device fabrication, as shown in Figure S2b, indicating that the devices are robust. Further, the retention of the HRS and LRS of the HfO_*x*_/SiO_*x*_ barrier layer device was also measured. As shown in Figure 3f, the HRS and LRS states can be retained for a long time (>10^4^ s) and are comparable to the baseline HfO_*x*_ device (Figure S3).

**Figure 4:**
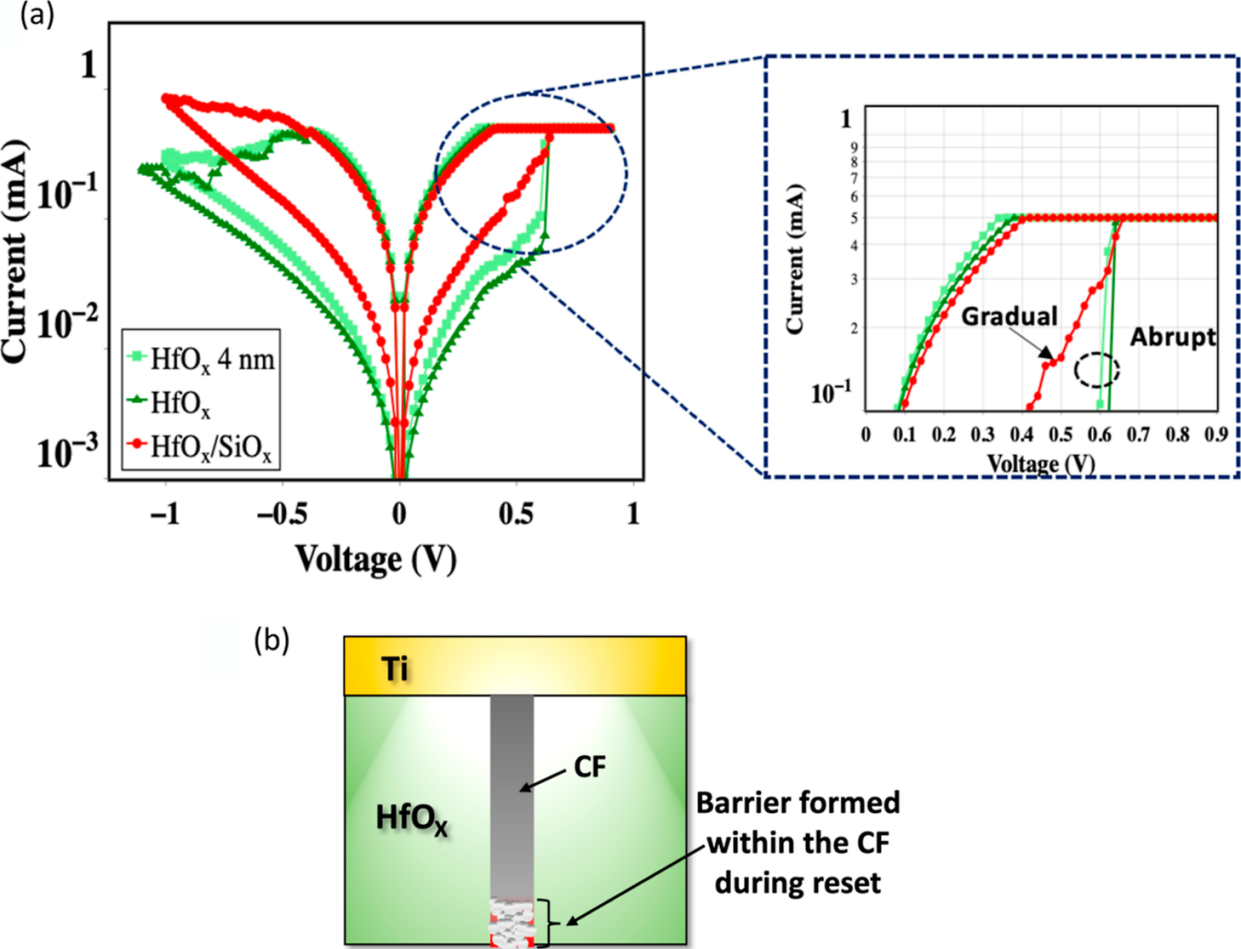
(a) Switching characteristics of 4 nm HfO_*x*_, 5 nm HfO_*x*_ device, and HfO_*x*_/SiO_*x*_ device. The 4 nm HfO_*x*_ has a small switching window compared to 5 nm HfO_*x*_. Similar to 5 nm HfO_*x*_, the set side resistance transition in 4 nm HfO_*x*_ is abrupt and is very different from HfO_*x*_/SiO_*x*_. (b) Dielectric barrier forms within the conductive filament during reset which determines the on/off ratio.

It is possible that the forming and switching characteristics of the HfO_*x*_/SiO_*x*_ (4 nm/1 nm) stacked device is dominated by the 4 nm HfO_*x*_ layer. To evaluate this possibility, the forming and switching characteristics of a device with only 4 nm HfO_*x*_ were analyzed. The 4 nm HfO_*x*_ device has a smaller forming voltage (Figure S4) and switching window (Figure 4a) as compared to the 5 nm HfO_*x*_ device. This relationship between the dielectric thickness and device characteristics in HfO_*x*_ is expected and has previously been demonstrated.^62^ Furthermore, the set transition for the 4 nm HfO_*x*_ device is abrupt, which indicates that the reduction of the HfO_*x*_ dielectric thickness to 4 nm is not the sole cause for the observed switching characteristics in the HfO_*x*_/SiO_*x*_ devices. The forming and switching characteristics of a 5 nm SiO_*x*_ device were also evaluated, as shown in Figure S5. The SiO_*x*_ device shows a much higher forming voltage compared to the HfO_*x*_ device and a significantly different forming characteristics, and a smaller switching window compared to the HfO_*x*_/SiO_*x*_ device. The switching characteristics of 5 nm SiO_*x*_ are similar to a prior work,^63^ indicating that the inherent switching mechanism of 5 nm SiO_*x*_ differs from the HfO_*x*_/SiO_*x*_ (4 nm/1 nm) stacked device. A combination of HfO_*x*_ and SiO_*x*_ is responsible for the observed change in switching characteristics of HfO_*x*_/SiO_*x*_ (4 nm/1 nm) stacked devices.

Although controlled resistance change during the set is achieved by adding the SiO_*x*_ barrier layer, the on–off resistance ratio (on–off ratio) is decreased compared to the HfO_*x*_ device. The reduction of the on–off ratio is caused by a reduction of the HRS, as shown in Figure 3b. The on–off ratio is determined by the oxide region, which is formed inside the filament during reset, as shown in Figure 4b. An analysis of the reset side would facilitate a deeper understanding of the oxide region formed within the conductive filament. Therefore, a finite element analysis (FEA) simulation is performed at the reset side.

**Figure 5:**
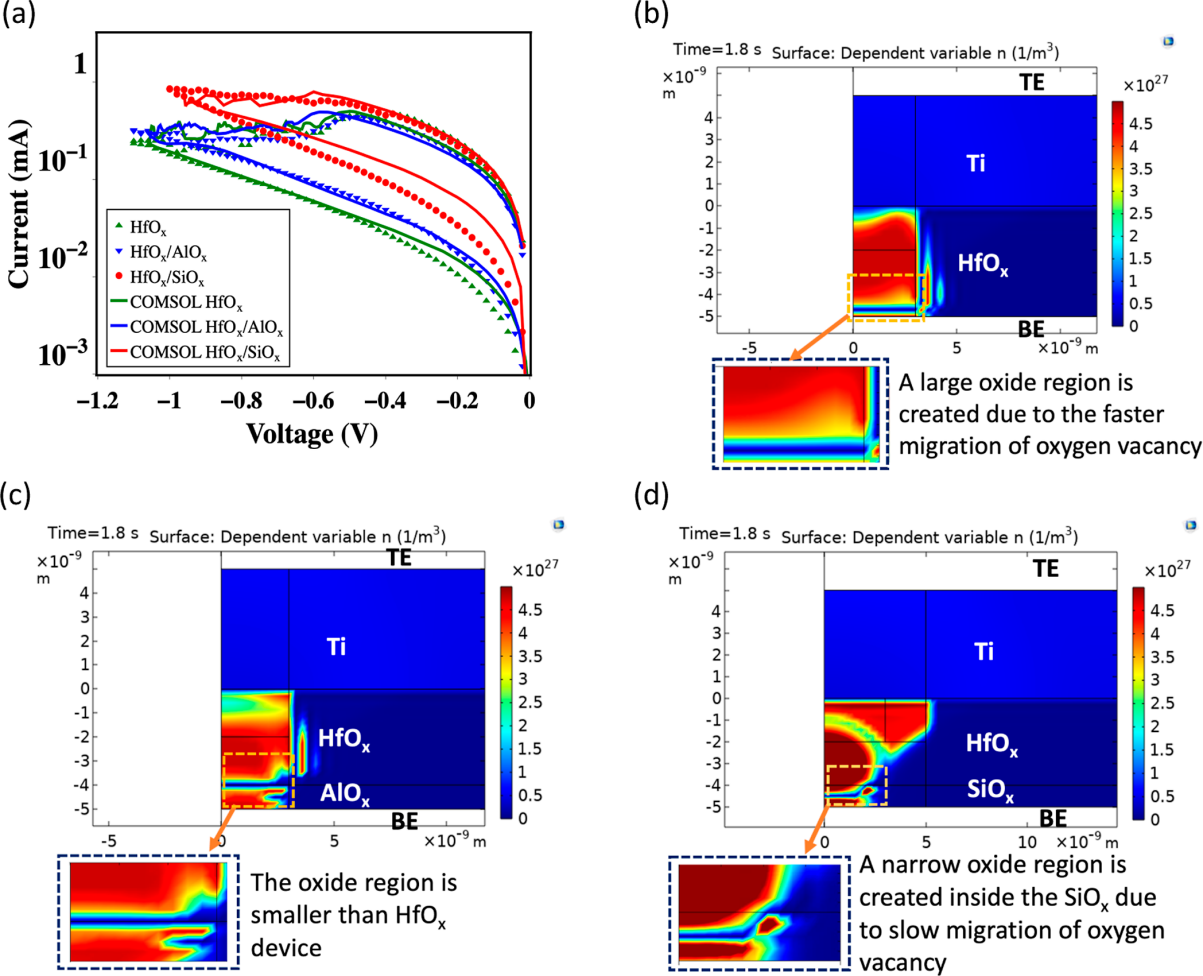
(a) FEA simulation of the current–voltage relationship in the reset transition. The simulated *I*–*V* shows a trend similar to the experimental *I*–*V*. The simulation results show that the filament breaks upon the formation of oxide in the reset in (b) HfO_*x*_, (c) HfO_*x*_/AlO_*x*_, and (d) HfO_*x*_/SiO_*x*_ devices. The oxide region is largest for the HfO_*x*_ device and smallest for the HfO_*x*_/SiO_*x*_ device. The oxide region decreases with the increase of the activation energy of the *V*_o_ diffusion.

Figure 5 shows the reset side of the current–voltage relationship resulting from the FEA modeling. The initial state of the simulation considers a fully formed filament connecting the top and the bottom electrodes. The initial filament shape is defined by piecewise functions with different equations for different segments along each axis. The functions used to determine the filament shape are elaborated in the Supporting Information (Figure S6). The resistive switching process of the devices is governed by three factors: *V*_o_ concentration gradient, local electric field, and local thermal field imposed by Joule heating. Ielmini et al.^64^ proposed that the switching process can be realized by self-consistently solving three main governing equations: (1) drift, diffusion continuity equation, (2) current conservation equation, and (3) Fourier Joule heating equation, as shown in eq S1 to eq S3, respectively. We have recently developed^27^ an FEA model which self-consistently solves these three partial differential equations (PDEs) with additional equations defining the electrical conductivity (σ), thermal conductivity (*k*), diffusion coefficient (*D*_*v*_), thermophoresis co-efficient (*S*_*v*_), and oxygen vacancy drift velocity (*υ*_*υ*_) (eq S4, to eq S8). The FEA simulation determines the *V*_o_ concentration (*n*_*v*_), electric potential (ψ), and local temperature (*T*) as a function of device spatial co-ordinate. The details of the model parameters can be found in Table S1.

Figure 5a shows the simulated *I*–*V* characteristics on the reset side. The simulated current–voltage shows good agreement with the experimental data. It is observed that the off-resistance state during reset decreases with the increase of the *V*_o_ migration barrier in the barrier layer, which is similar to the experimental data. Figure 5b–d shows the cross-sectional view of the filaments of HfO_*x*_, HfO_*x*_/AlO_*x*_, and HfO_*x*_/SiO_*x*_ devices. In HfO_*x*_ devices, the oxide region (break area) in the filament during the reset is the largest. The break region decreases as the migration barrier of *V*_o_ goes high for HfO_*x*_/AlO_*x*_ and HfO_*x*_/SiO_*x*_ devices. The HfO_*x*_/SiO_*x*_ device has the narrowest break region. The thin oxide reduces the off-resistance state, thus lowering the on–off ratio. The analysis shows that the oxide area formed during reset decreases with increasing the *V*_o_ migration barrier at the bottom electrode/oxide interface.

**Figure 6:**
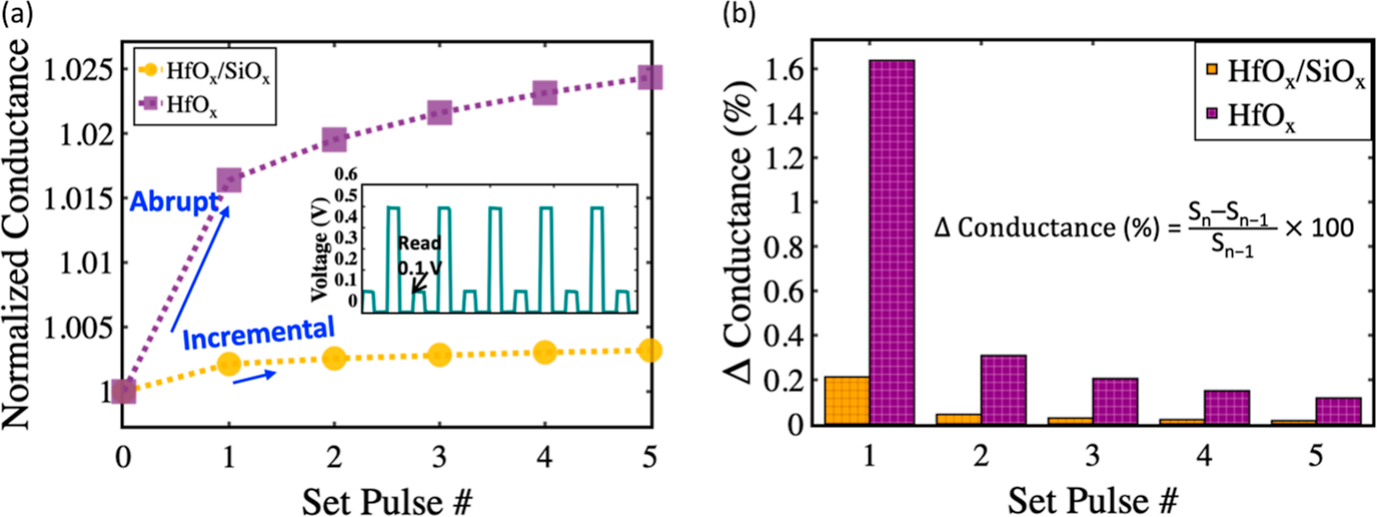
(a) Simulated normalized conductance change vs pulse number. Upon application of positive set pulses, the HfO_*x*_ device exhibits an abrupt conductance increase, whereas the HfO_*x*_/SiO_*x*_ device has a gradual increase in conductance, as depicted in the graph. (b) Percentage of conductance change with respect to set pulses. The HfO_*x*_/SiO_*x*_ device has a relatively small conductance change (0.21% at the first pulse), while the HfO_*x*_ device exhibits a significantly higher conductance change (1.64% at the first pulse), which is ∼8 times greater than that of the HfO_*x*_/SiO_*x*_ device. Here, the normalized conductance is defined as the conductance divided by the minimum conductance. The percentage conductance change is defined as the ratio of the change in conductance at the current pulse and the conductance at the previous pulse to the conductance at the previous pulse.

Furthermore, analog pulses are employed in the COMSOL simulation to validate the gradual conductance increase of the HfO_*x*_/SiO_*x*_ devices. Specifically, when positive pulses (pulse width 0.5 V, amplitude 1 ms, Figure S7) are applied, the HfO_*x*_ device displays an abrupt conductance increase. In contrast, the HfO_*x*_/SiO_*x*_ device shows a gradual increase in conductance, as demonstrated in Figure 6a. Experimental results further supported this observation, indicating that the conductance change in the HfO_*x*_/SiO_*x*_ device is gradual compared to that of the HfO_*x*_ device (Figure S8). Additionally, Figure 6b presents the percentage of conductance change data with respect to set pulses. It can be observed that the HfO_*x*_/SiO_*x*_ devices exhibit a small change in conductance (0.21% at the first pulse), while the HfO_*x*_ device has a significantly higher conductance change (1.64% at the first pulse), which is ∼ eight times greater than that of the HfO_*x*_/SiO_*x*_ device.

**Figure 7:**
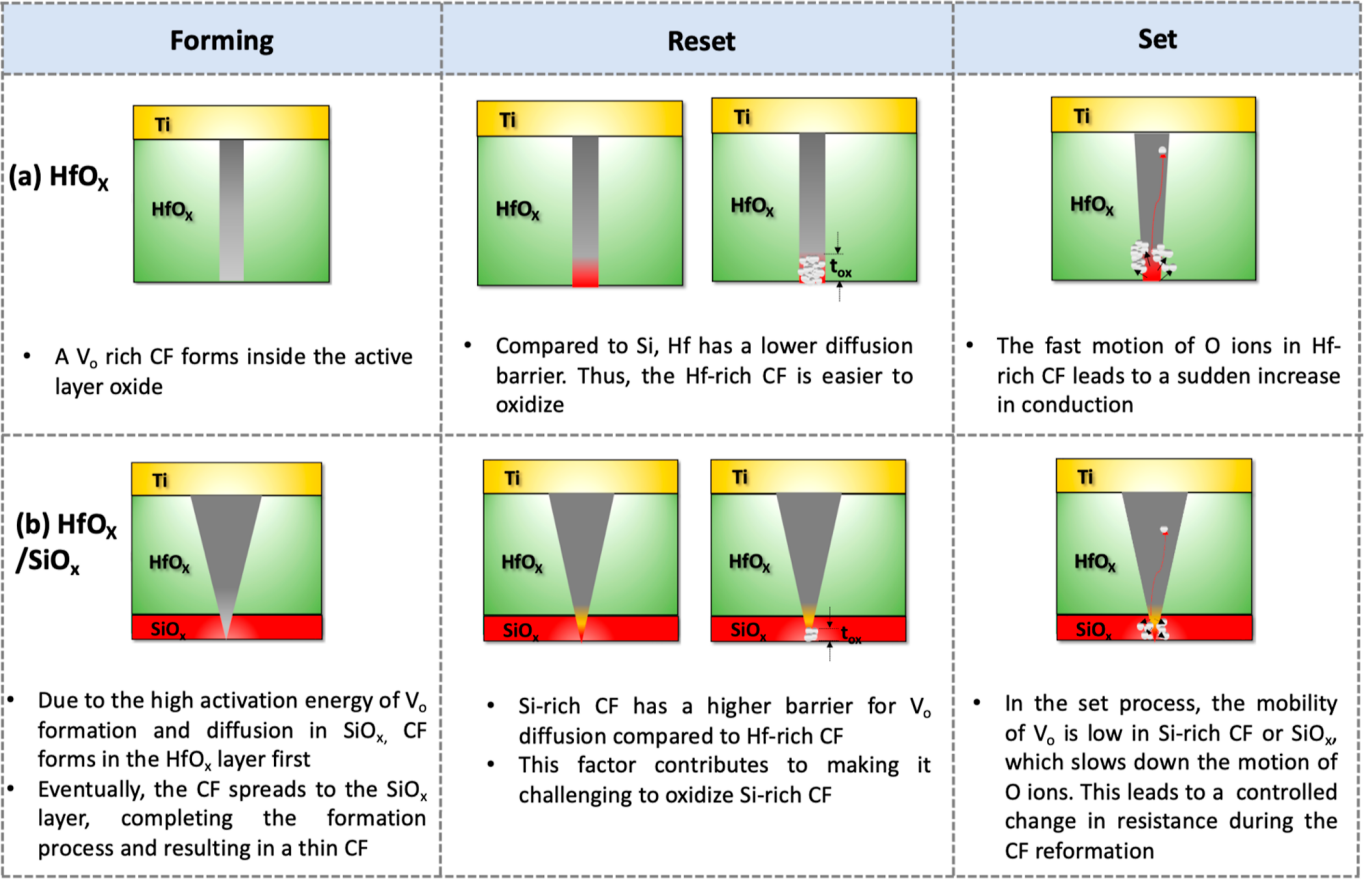
Possible switching mechanism in (a) HfO_*x*_ and (b) HfO_*x*_/SiO_*x*_ devices. Due to the low diffusion barrier of *V*_o_ in HfO_*x*_, a thick oxide is formed in the filament during reset, and subsequently, abrupt oxygen ion motion occurs during the set. In contrast, the high *V*_o_ diffusion barrier in the HfO_*x*_/SiO_*x*_ device causes the formation of a thin oxide during the reset, and oxygen ions are gradually removed during set.

Figure 7 depicts the proposed mechanism for the aforementioned devices. At first, during formation, the filament shape is modulated in the stacks because of the difference in *V*_o_ formation energy (HfO_2_ = 4.12–4.4 eV,^42,65^ SiO_2_ = 5.16–8.1 eV)^66,67^ and higher activation energy for oxygen vacancy diffusion in SiO_2_ (∼4.6 eV)^44,48^ compared to HfO_2_ (∼1.5 eV).^34^ In a HfO_*x*_/SiO_*x*_ device, the *V*_o_ formation and diffusion barrier in HfO_*x*_ is lower than SiO_*x*_. Initially, the formation of the *V*_o_-rich filament starts in the HfO_*x*_ layer, where the *V*_o_ formation and diffusion barrier are lower. Then the filament extends to the SiO_*x*_ layer, finishing the forming process and resulting in a thick section in HfO_*x*_ and a thin section in SiO_*x*_, compared to the HfO_*x*_ device.

During reset, as the negative bias is applied at the top electrode, the filament breaks at the bottom electrode (anode)/oxide interface.^34,42^ In the HfO_*x*_ device, due to a low migration barrier of *V*_o_, oxide formation is easier. Therefore, a large oxidized region in the filament is created. During the set, the oxygen ion removal is fast, and the sudden removal of oxygen ions results in an abrupt set. On the other hand, in the HfO_*x*_/SiO_*x*_ device, the Si-rich filament has a high *V*_o_ diffusion barrier. This factor makes the oxide formation challenging and result in the creation of a narrow oxide region within the filament, leading to a reduction in the off-resistance state. During set, because of the high *V*_o_ migration barrier, the oxygen ion removal process is slow, resulting in controlled resistance change.

## 4 Conclusions

A thin (∼1 nm) interfacial barrier layer (SiO_*x*_, AlO_*x*_) at the bottom electrode/oxide interface is added to enable better control over the *V*_o_’s motion during the set and evaluate its impact on the on–off ratio in two terminal HfO_*x*_ memristors. Among the fabricated devices, the HfO_*x*_/SiO_*x*_ device shows controlled resistance change in the set because of the slow motion of *V*_o_. The device-to-device and cycle-to-cycle variability of resistance change is observed to be better in the HfO_*x*_/SiO_*x*_ device compared to baseline HfO_*x*_ devices. Moreover, the FEA COMSOL Multiphysics simulation further demonstrates that the formed oxide region in the conductive filament decreases with the increase of migration barrier at the BE/oxide interface. The high diffusion barrier of SiO_*x*_ results in less abrupt resistance changes during the set, less variation in resistance changes, and an on–off ratio of ∼10, which are sufficient for many analog neuromorphic applications.^68,69^ Future experimental analysis, such as electron energy loss spectroscopy (EELS), is necessary to observe oxygen vacancy distribution and understand the reason for less variability in the HfO_*x*_/SiO_*x*_ devices. Unlike non-CMOS compatible or complex device structure-based approaches, the simple CMOS-friendly SiO_*x*_ barrier layer concept can be easily adopted at the industry level to achieve controlled resistance changes and less variability, which are required for deep-learning applications like training and transfer learning of large-scale artificial neural networks.

## Supporting Information Available

Additional discussions about the detail of device dimensions, 4 nm HfO_*x*_ and 5 nm SiO_*x*_ device resistive switching characteristics and retention, and details of the COMSOL Multiphysics simulation (PDF)
