# Supplementary material for: Trade-off between Gradual Set and On/Off Ratio in HfOx-Based Analog Memory with a Thin SiOx Barrier Layer
Source: ACS Appl Electron Mater. 2023 Jun 1;5(6):3048–58. doi: 10.1021/acsaelm.3c00131 (PMC10308818; doi:10.1021/acsaelm.3c00131)
Supplement: Supplementary file 1 — el3c00131_si_001.pdf [file el3c00131_si_001.pdf]

(a) (b)

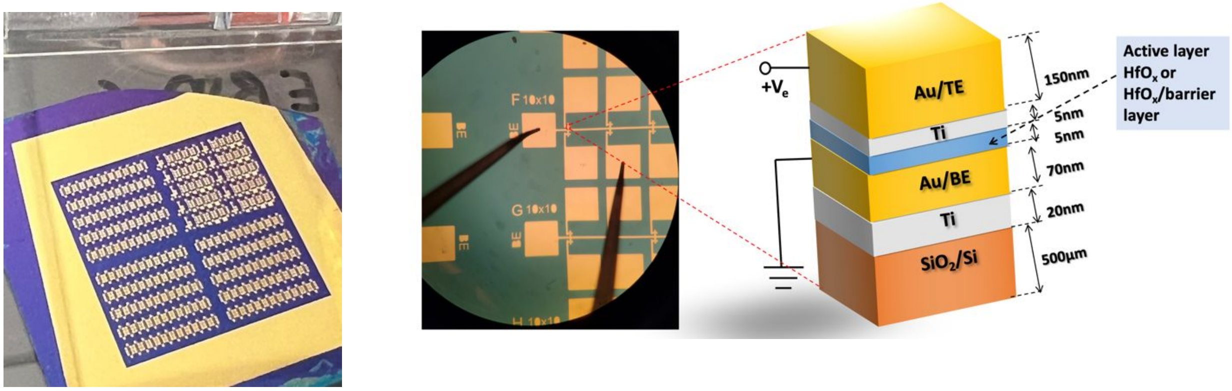

Supporting Information

The Trade-off Between Gradual Set and On/Off ratio in HfO_x-based Analog Memory with a Thin SiO_x Barrier Layer

Fabia F. Athena[1], Matthew P. West[2], Jinho Hah[2], Samuel Graham[3,4] and Eric M. Vogel[1,2]*

[1]School of Electrical and Computer Engineering, Georgia Institute of Technology, Atlanta, Georgia 30332, USA

[2]School of Materials Science and Engineering, Georgia Institute of Technology, Atlanta, Georgia 30332, USA

[3]Department of Mechanical Engineering, University of Maryland, College Park, Maryland 20742, USA

[3]George W. Woodruff School of Mechanical Engineering, Georgia Institute of Technology, Atlanta, Georgia 30332, USA

[*]Email: eric.vogel@mse.gatech.edu

(c)

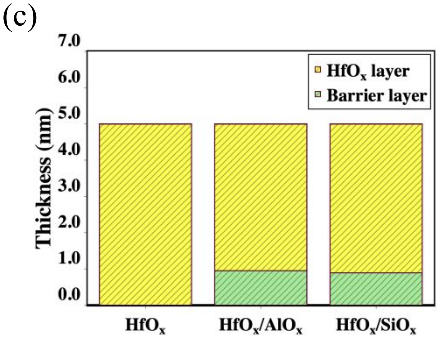

Figure S1: (a) Optical image of a representative sample device. The device size is 10x10 μm^2 (b) Top and bottom electrode connections during forming and set. The schematic illustration showing the full device stack showing the dimensions of different sections. (c) Average ellipsometry measurements shows that the barrier layer thickness is around ~1 nm in both HfO_x/AlO_x and HfO_x/SiO_x device. This also indicates uniform deposition of the ALD oxide film.

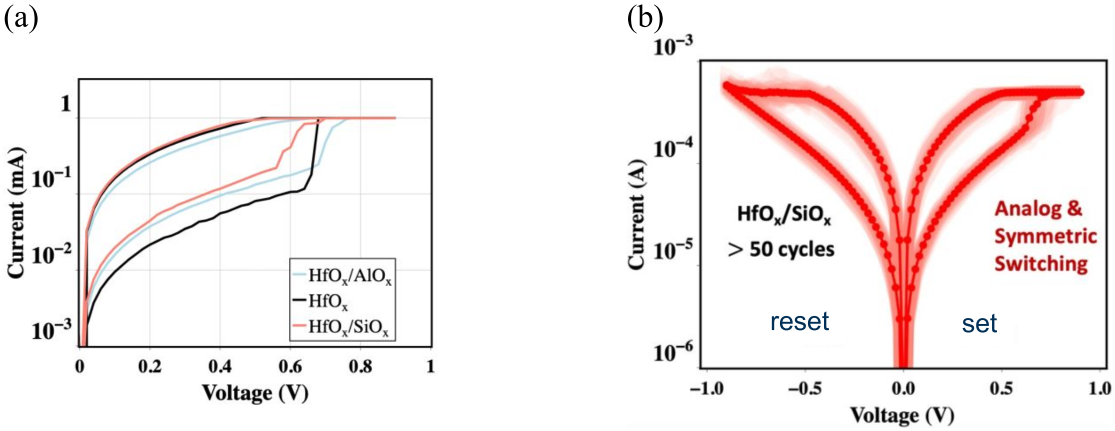

Figure S2. (a) The resistance changes from HRS to LRS at high current compliance (~1 mA) of HfO_x, HfO_x/AlO_x, and HfO_x/SiO_x devices. At high current compliance resistance change is less abrupt for HfO_x/SiO_x device compared to other two. (b) The DC I-V characteristics of HfO_x/SiO_x device under ± 0.9 V sweeping voltage with current compliance 0.5 mA measured after ~6000 hours. The switching characteristics in HfO_x/SiO_x is symmetric and analog and is retained even after 6000 hrs.

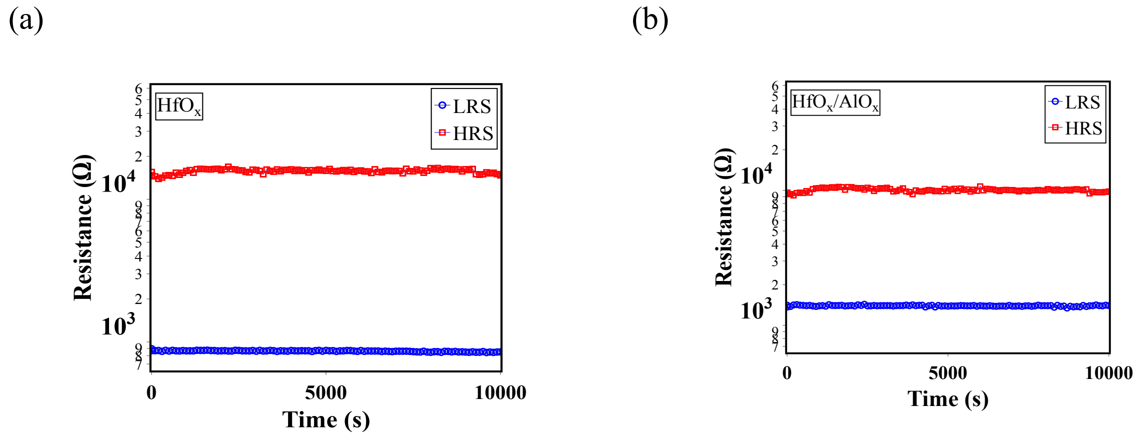

Figure S3. Retention characteristics of (a) HfO_x, (b) HfO_x/AlO_x. Retention characteristics of HfO_x/SiO_x barrier layer device is comparable to the control device.

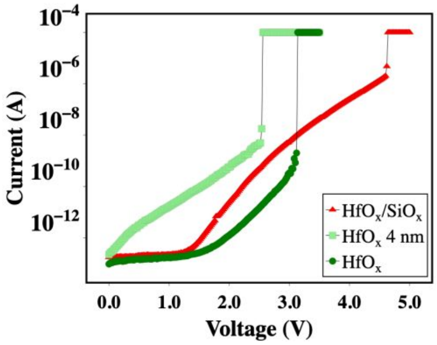

Figure S4. Forming characteristics comparison in 4 nm HfO_x, 5 nm HfO_x device and HfO_x/SiO_x device. 4 nm HfO_x device has low forming voltage and similar forming characteristics to 5 nm HfO_x device.

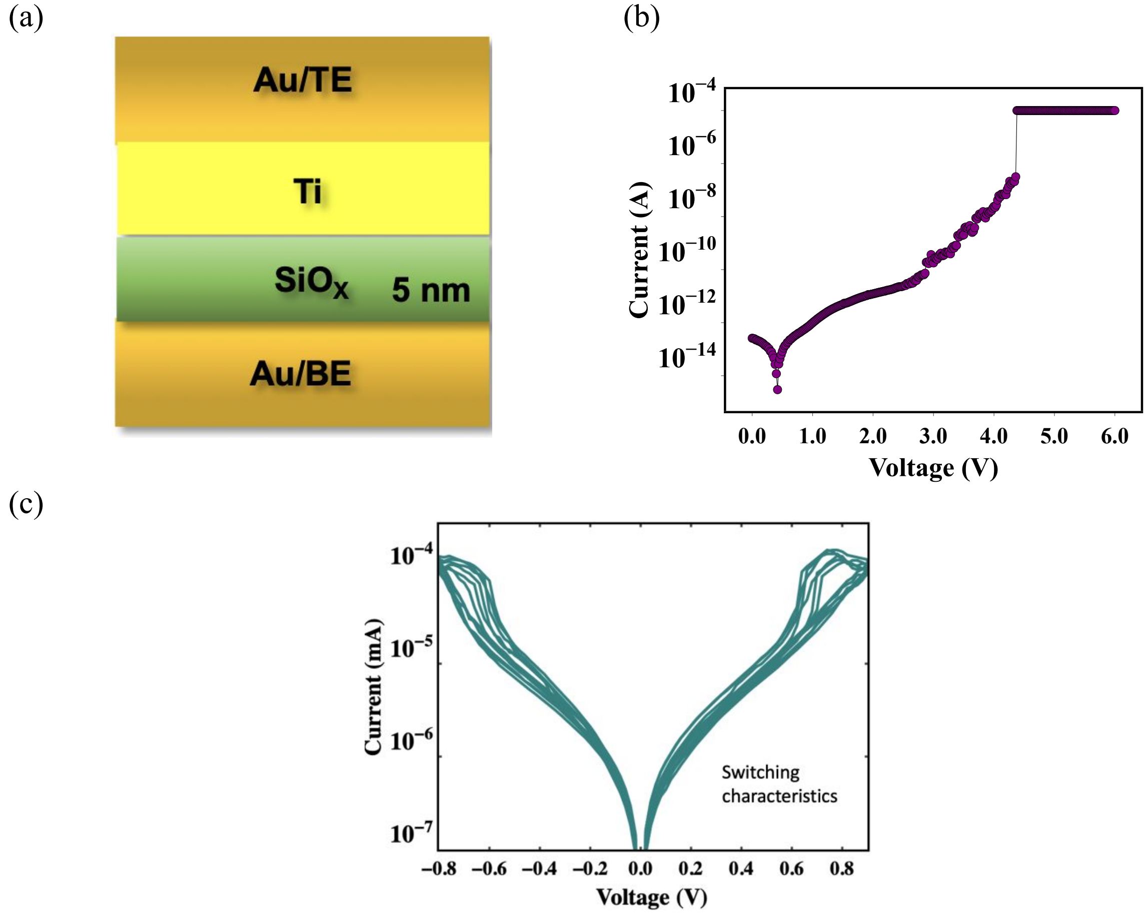

Figure S5. (a) Schematics of 5 nm SiO_x device. (b) Forming characteristics of 5 nm SiO_x device. (c) Switching characteristics in 5 nm SiO_x device. The switching characteristics is significantly different from the HfO_x/SiO_x device.

Details of the FEA model:

$$\frac{\partial n_v}{\partial t} = -\nabla(v_v \cdot n_v) + \nabla \cdot (D_v \nabla n_v) + \nabla \cdot (S_v \cdot D_v \cdot n_v \nabla T) \qquad (1)$$

$$\nabla \cdot (\sigma \nabla \psi) = 0 \qquad (2)$$

$$\frac{\partial T}{\partial t} = \frac{1}{\rho c} \nabla \cdot (kT) + \frac{\sigma}{\rho c} (\nabla \psi)^2 \qquad (3)$$

Here n_v is oxygen vacancy density, v_v is drift velocity of oxygen vacancies, D_v is diffusion coefficient of the oxygen vacancy, S_v is the thermophoresis coefficient, e is permittivity of the oxide material, T is temperature, ρ is density, Cp is specific heat capacity, k_{th} is thermal conductivity. In three different overlapping domains these three equations are solved. The vacancy conservation equation was solved for Titanium capping layer and HfO_x domain. As the gold electrodes don't interact with vacancies, the boundary for vacancy conservation was considered up to the capping layer and active layer. The current conservation equation was solved from the top gold electrode to bottom gold electrode. The bias was applied on the top electrode and the bottom electrode was grounded. The rest of the boundaries of the domain were electrically insulating. The energy conservation equation was solved across the whole geometry including the substrate. The substrate bottom was fixed at 22^0C and was considered as in perfect contact with an infinite heat sink. The thermal conductivity, electrical conductivity, thermophoresis coefficient and oxygen vacancy drift velocity were derived from eq. (4), (5), (6), (7), and (8).

$$\sigma = \frac{(\sigma_{HfO_x} - \sigma_{HfO_2})}{n_{V,Max}} \cdot n_V e^{-\frac{E_{AC}}{k_B T}} \qquad (4)$$

$$k = k_{HfO_2} + \frac{(k_{Hf} - k_{HfO_2})}{n_{V,Max}} \cdot n_V \qquad (5)$$

$$D_v = \frac{fa^2}{2} \cdot e^{-\frac{E_a}{k_B T}} \qquad (6)$$

$$S_v = -\frac{E_a}{k_B T^2} \qquad (7)$$

$$vv = f.a.e^{(-\frac{E_a}{k_B T})} . \sinh(\frac{-qa\nabla\psi}{kBT}) \qquad (8)$$

Here thermophysical property σ and k vary as oxygen vacancy concentration changes. Here, k_B is Boltzmann constant, E_a is activation energy for oxygen vacancy diffusion, a is the lattice parameter. Table S1 provides activation energy values for oxygen vacancy diffusion utilized in the simulation. While there is a range in general for the activation energy of neutral and charged oxygen vacancy diffusion, it is higher in SiO_x (2.03 – 4.6 eV)[1-4] compared to AlO_x (1.26 – 3.6

eV)[5, 6] and HfO_x (0.7 — 1.5 eV)[7-9] We found that the values presented in Table S1 align most closely with the experimental results. In particular, higher activation energy values are used in the SiO_x (2.2 eV) and AlO_x (1.58 eV) layers of the HfO_x/SiO_x and HfO_x/AlO_x devices compared to the HfO_x (1.5 eV) device. The same activation energy value (1.5 eV) was used in the HfO_x layer of the HfO_x/SiO_x and HfO_x/AlO_x devices.

Table S1: The numerical values of the parameters used in the model

Parameter	Value
$n_{V\ Max}$	4.85 x 10^{27} 1/m^3
Ea HfO_x	1.5 eV
Ea SiO_x	2.2 eV
Ea AlO_x	1.58 eV
Do	5e-6 cm^2/s
$E_{AC,\ Max}$	0.36 eV
$\boldsymbol{\rho}$	9680 kg/m^3
k_{HfOx}	23 W/m.K

The values of other important parameters used in the COMSOL Multiphysics® simulation can be found in our previously published study by Pahinkar *et al.*[9]

For the filament shape three representative equations can be used such as step change, exponential decay and hyperbolic tangent. The combination of these three profiles resulted in desired conical filament shape for HfO_x/SiO_x case, which also resulted in better model validation.

(a)

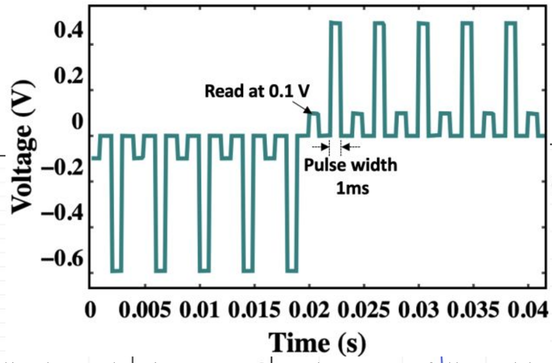

Figure S7. Applied pulse amplitude vs the time. 5 reset pulses were followed by 5 set pulses. The conductance during the set pulse was calculated from the read pulse (amplitude 0.1 V). Identical pulses are used during the analog measurement.

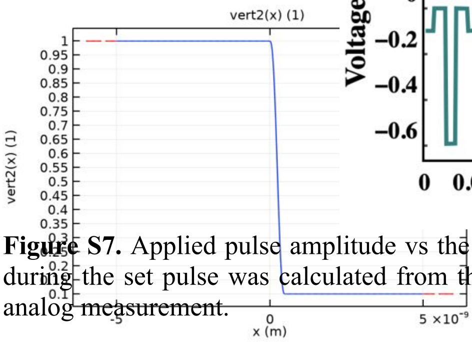

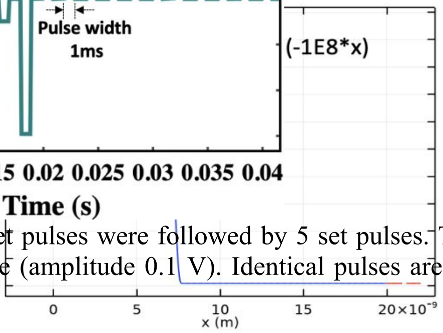

(c)

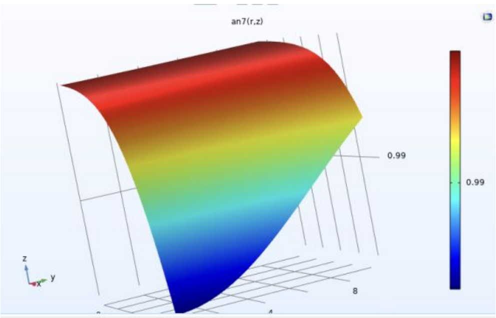

Figure S6. (a) Piecewise step function to determine the boundary of the filament. (b) horizontal distribution to define the horizontal exponential decay of vacancy concentration of the filament. (c) hyperbolic tangent to define the shape.

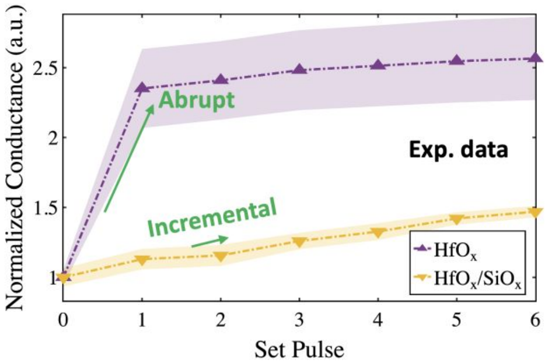

Figure S8. The normalized conductance change under positive set pulses, measured experimentally. The conductance change at the first pulse is abrupt in the HfO_x device compared to the HfO_x/SiO_x device. Pulse amplitude in HfO_x is 0.6 V, 1ms and for HfO_x/SiO_x is 0.5 V, 1 ms. The optimum pulse amplitudes are used for each device to achieve the maximum switching window and small linearity factor, also known as alpha[6]) value. The normalized conductance is determined by diving the conductance at each point by the minimum conductance. The shaded region corresponds to the 95% confidence interval[7, 8] from the measured (~7) devices.

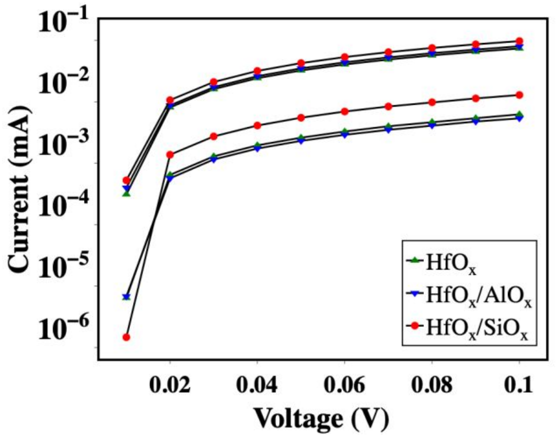

Figure S9. Average current response during the application of small I-V sweeps (0.6 V) for the measurement of the HRS and LRS. To measure the LRS, a small 0.1 V sweep was applied after each set sweep under the positive bias. Likewise, a small 0.1 V sweep was applied after each reset sweep to measure the HRS of the device.

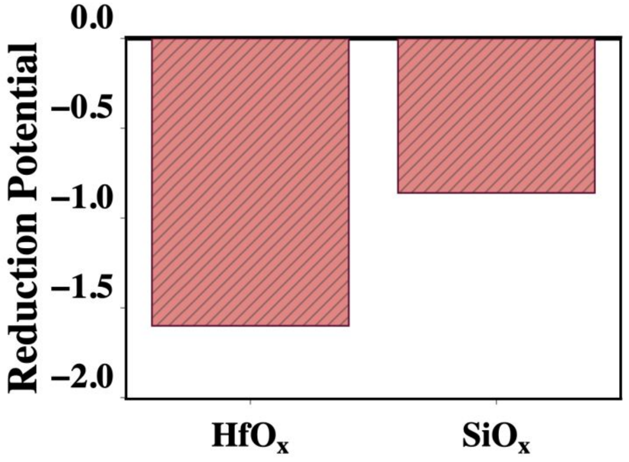

Figure S10. Reduction potential in HfO_x and SiO_x. Reduction potential is more positive in SiO_x compared to HfO_x. Larger negative reduction potential in baseline HfO_x device makes oxide formation easier during reset compared to HfO_x/SiO_x device.

References for Supporting Information:

(1) Mehonic, A.; Shluger, A. L.; Gao, D.; Valov, I.; Miranda, E.; Ielmini, D.; Bricalli, A.; Ambrosi, E.; Li, C.; Yang, J. J. Silicon oxide (SiOx): a promising material for resistance switching? *Advanced materials* **2018**, *30* (43), 1801187.
(2) Munde, M. S.; Gao, D. Z.; Shluger, A. L. Diffusion and aggregation of oxygen vacancies in amorphous silica. *Journal of Physics: Condensed Matter* **2017**, *29* (24), 245701.
(3) Cho, Y. J.; Sun, K.; Was, G.; Lu, K. In-situ microstructure observation of oxidized SiC layer in surrogate TRISO fuel particles under krypton ion irradiation. *Journal of Alloys and Compounds* **2022**, *920*, 165833.
(4) You, N.; Liu, X.; Zhang, Q.; Xu, Y.; Wang, J.; Wang, S. Oxygen diffusion kinetics during SiO2/SiC plasma oxidation. *Vacuum* **2023**, *207*, 111689.
(5) Carrasco, J.; Lopez, N.; Illas, F. First principles analysis of the stability and diffusion of oxygen vacancies in metal oxides. *Physical review letters* **2004**, *93* (22), 225502.
(6) Yang, M. Y.; Kamiya, K.; Magyari-Köpe, B.; Niwa, M.; Nishi, Y.; Shiraishi, K. Charge-dependent oxygen vacancy diffusion in Al2O3-based resistive-random-access-memories. *Applied Physics Letters* **2013**, *103* (9), 093504.
(7) Bersuker, G.; Gilmer, D.; Veksler, D.; Kirsch, P.; Vandelli, L.; Padovani, A.; Larcher, L.; McKenna, K.; Shluger, A.; Iglesias, V. Metal oxide resistive memory switching mechanism based on conductive filament properties. *Journal of Applied Physics* **2011**, *110* (12), 124518.
(8) Larcher, L.; Padovani, A.; Pirrotta, O.; Vandelli, L.; Bersuker, G. Microscopic understanding and modeling of HfO 2 RRAM device physics. In *2012 International Electron Devices Meeting*, 2012; IEEE: pp 20.21. 21-20.21. 24.
(9) Pahinkar, D. G.; Basnet, P.; West, M. P.; Zivasatienraj, B.; Weidenbach, A.; Doolittle, W. A.; Vogel, E.; Graham, S. Experimental and computational analysis of thermal environment in the operation of HfO2 memristors. *AIP Advances* **2020**, *10* (3), 035127.
